# Targeted DNA-seq and RNA-seq of Reference Samples with Short-read and Long-read Sequencing

**DOI:** 10.1038/s41597-024-03741-y

**Published:** 2024-08-16

**Authors:** Binsheng Gong, Dan Li, Paweł P. Łabaj, Bohu Pan, Natalia Novoradovskaya, Danielle Thierry-Mieg, Jean Thierry-Mieg, Guangchun Chen, Anne Bergstrom Lucas, Jennifer S. LoCoco, Todd A. Richmond, Elizabeth Tseng, Rebecca Kusko, Scott Happe, Timothy R. Mercer, Carlos Pabón-Peña, Michael Salmans, Hagen U. Tilgner, Wenzhong Xiao, Donald J. Johann, Wendell Jones, Weida Tong, Christopher E. Mason, David P. Kreil, Joshua Xu

**Affiliations:** 1 Division of Bioinformatics and Biostatistics, National Center for Toxicological Research, US Food and Drug Administration, Jefferson, AR 72079 USA; 2 Małopolska Centre of Biotechnology, Jagiellonian University, Krakow, Poland; 3 Bioinformatics Research, Institute of Molecular Biotechnology, Boku University Vienna, Vienna, Austria; 4 Agilent Technologies, Inc., 11011 N Torrey Pines Rd., La Jolla, CA 92037 USA; 5 National Center for Biotechnology Information, National Library of Medicine, National Institutes of Health, 8600 Rockville Pike, Bethesda, MD 20894 USA; 6 Department of Immunology, Genomics and Microarray Core Facility, University of Texas Southwestern Medical Center, 5323 Harry Hine Blvd., Dallas, TX 75390 USA; 7 Agilent Technologies, Inc., 5301 Stevens Creek Blvd., Santa Clara, CA 95051 USA; 8 Illumina Inc., 5200 Illumina Way, San Diego, CA 92122 USA; 9 Market & Application Development Bioinformatics, Roche Sequencing Solutions Inc., 4300 Hacienda Dr., Pleasanton, CA 94588 USA; 10 PacBio, San Francisco, USA; 11 Cellino Bio, 750 Main Street, Cambridge, MA 02143 USA; 12 Agilent Technologies, Inc., 1834 State Hwy 71 West, Cedar Creek, TX 78612 USA; 13 Australian Institute for Bioengineering and Nanotechnology, The University of Queensland, St Lucia, QLD, Australia; 14 Brain and Mind Research Institute, Weill Cornell Medicine, New York, NY USA; 15 Center for Neurogenetics, Weill Cornell Medicine, New York, NY USA; 16 Stanford Genome Technology Center, Stanford University, Palo Alto, CA 94304 USA; 17 Massachusetts General Hospital, Harvard Medical School, Boston, MA 02114 USA; 18 Winthrop P Rockefeller Cancer Institute, University of Arkansas for Medical Sciences, 4301W Markham St., Little Rock, AR 72205 USA; 19 Q squared Solutions Genomics, 2400 Elis Road, Durham, NC 27703 USA; 20 Department of Physiology and Biophysics, Weill Cornell Medicine, Cornell University, New York, NY 10065 USA; 21 The HRH Prince Alwaleed Bin Talal Bin Abdulaziz Alsaud Institute for Computational Biomedicine, Weill Cornell Medicine, New York, NY USA; 22 The WorldQuant Initiative for Quantitative Prediction, Weill Cornell Medicine, New York, NY USA

**Keywords:** Next-generation sequencing, Data publication and archiving, Research data

## Abstract

Next-generation sequencing (NGS) has revolutionized genomic research by enabling high-throughput, cost-effective genome and transcriptome sequencing accelerating personalized medicine for complex diseases, including cancer. Whole genome/transcriptome sequencing (WGS/WTS) provides comprehensive insights, while targeted sequencing is more cost-effective and sensitive. In comparison to short-read sequencing, which still dominates the field due to high speed and cost-effectiveness, long-read sequencing can overcome alignment limitations and better discriminate similar sequences from alternative transcripts or repetitive regions. Hybrid sequencing combines the best strengths of different technologies for a more comprehensive view of genomic/transcriptomic variations. Understanding each technology’s strengths and limitations is critical for translating cutting-edge technologies into clinical applications. In this study, we sequenced DNA and RNA libraries of reference samples using various targeted DNA and RNA panels and the whole transcriptome on both short-read and long-read platforms. This study design enables a comprehensive analysis of sequencing technologies, targeting protocols, and library preparation methods. Our expanded profiling landscape establishes a reference point for assessing current sequencing technologies, facilitating informed decision-making in genomic research and precision medicine.

## Background & Summary

Next-generation sequencing is a powerful technology that has ushered in a Cambrian era of genomic research by enabling high-throughput, cost-effective DNA and RNA sequencing. DNA sequencing of entire genomes, exomes, or targeted regions can help pinpoint genetic variations, mutations, and other genomic changes^1^. RNA sequencing of whole transcriptomes (WTS) or a targeted set of transcripts can provide insight into gene expression, alternative splicing, gene fusions, RNA editing, and identify novel transcripts^2^. Over the past decades, NGS technologies have been extensively leveraged to make significant discoveries spanning a wide range of research areas, including complex diseases like cancer and revolutionizing clinical applications for personalized medicine and more^3–5^.

Whole genome/transcriptome sequencing can provide a comprehensive view of the entire genome/transcriptome and hypothesis-free discovery, allowing a wide range of applications, including splicing or sequence variant detection, genome assembly, biomarker discovery, etc^6^. Targeted sequencing, on the other hand, is often more cost-effective and can provide higher accuracy and sensitivity via focused coverage of the genes or regions of interest, making it of great interest in a wide range of research and clinical settings^7^.

For many years, DNA sequencing has predominantly utilized short-read technology, due to its rapid, high-throughput, cost-effectiveness, and established workflows^8^. Short-read sequencing has been widely deployed in large-scale sequencing projects, such as the Human Genome Project and the 1000 Genomes Project. While being effective for small variant detection or gene level expression profiling, short-read sequencing has limited ability to resolve repetitive regions, phase haplotypes, determine or quantify alternative gene transcript isoforms, and identify structural variations. These challenges are particularly pronounced for non-model organisms or in cases where the reference genome is incomplete or inaccurate, including personal human genomes with substantial variation.

In recent years, long-read sequencing technologies have emerged as a complementary method. Long-read RNA sequencing has allowed the determination of expression levels of complete isoforms, whether already annotated or novel^9–11^, allele-specific isoform usage^12,13^, and the combination patterns of TSS, exons, and poly(A) sites^14–17^. More recently, applying long-read RNA sequencing to thousands of single cells has allowed the identification of cell-type specific TSS, and exon and poly(A) site usage in fresh tissues^18–20^ and frozen tissues^21^. The advent of direct RNA sequencing^22^ has opened the door to the analysis of RNA modifications with long-read sequencing methods^23–28^. Applications of long-read RNA sequencing have advanced the study of diseases, including cancer^29–32^ and viral research^33,34^. Various long-read sequencing approaches have enabled the investigation of a wide variety of basic-biology and disease-related questions, leading to long-read sequencing being hailed as the method of the year for 2022, as described in detail for the RNA side^35–37^, the DNA side^36–38^ as well as for microbial genomics^36^. These developments suggest that long-read analysis of transcriptomes will continue to increase in popularity due to its ability to map reads long enough to span complex regions. Therefore, while short-read sequencing is still dominant, long-read sequencing is becoming more widely used in various applications, including genome assembly, structural variation detection, and transcript isoform identification^11,28,37,39,40^.

Different NGS technologies can yield distinct results for the same biological sample due to variations in sequencing material, read length, throughput, error rate, bioinformatic processing, and other protocol properties. The U.S. Food and Drug Administration (FDA)-led Sequencing Quality Control Phase 1 (SEQC1) conducted an extensive characterization of the quantitative properties of RNA-seq across multiple platforms and protocols^41–43^. Phase 2 of this project (SEQC2)^1^ went further, augmenting NGS analysis to include DNA sequencing in various applications to characterize the strengths and limitations of primary and alternative sequencing protocols, comparing short- and long-read technologies and a range of targeted sequencing panels. Our benchmark study is critical for informed protocol selection and a reliable interpretation of results for the entire genomics community. To this end, we prepared DNA and RNA libraries of the same reference samples for sequencing using a selection of targeting panels and whole transcriptome preparation kits. We sequenced these libraries using both short-read and long-read sequencing platforms. This study design allows the assessment of technical variability from various perspectives. For example, short-read sequencing technologies such as Illumina sequencing and Ion Torrent sequencing of targeted DNA libraries can be used to identify single nucleotide variants (SNVs) and small indels with high accuracy^44^. In targeted RNA-Seq, the high on-target rate allows for selective signal strengthening of on-panel genes, enabling more accurate quantification and differential expression analysis on both gene and alternative transcript levels, with reduced sequencing depth. On the other hand, long-read sequencing technologies such as Pacific Biosciences (PacBio) and Oxford Nanopore Technologies (ONT) of whole transcriptome libraries provide more accurate detection of alternative splicing and gene fusions^32,45,46^. In addition, it allows a substantial expansion of the transcriptional landscape for the genes targeted, and can yield reliable quantification of the alternative gene transcript expression levels, especially for complex genes, such as many oncogenes. Hybrid sequencing, which combines short- and long-read technologies, can overcome the limitations of a single technology alone. For example, short-read sequencing can generally provide high coverage (and thus sensitivity), while long-read sequencing can provide more isoform information, enable phasing of variants and splicing variants. Moreover, this approach usually outperforms short-read sequencing in repetitive regions or for families of similar sequences. Furthermore, sequencing both RNA and DNA libraries can increase variant call confidence, as well as provide variant functional annotation by linking them to gene expression^47^. Importantly, this assessment of splicing and activity variations in expression profiles is of high value in its own right.

## Methods

### Study design

**Fig. 1:**
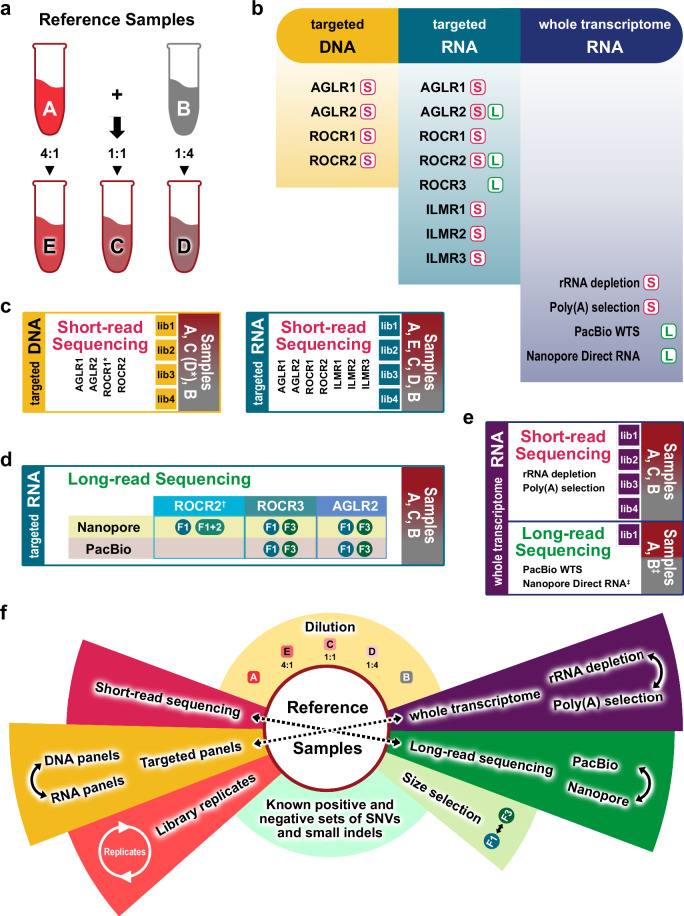
Illustration of study design. **(a)** To create reference samples C, D, and E: samples A and B were mixed in the ratios of 1:1, 1:4, and 4:1 respectively. **(b)** Three types of libraries were prepared for the reference samples: targeted RNA, targeted DNA, and whole transcriptome RNA. The libraries were sequenced using short-read or long-read sequencing methods, or both. The panel codes are explained in Table 2. The pink “S” represents short-read sequencing, while green “L” represents long-read sequencing. The ILMR3 panel is a whole exome RNA panel, and it was placed under “targeted RNA” for visual simplicity. **(c)** Both targeted DNA and targeted RNA libraries were sequenced with short-read sequencing. For targeted DNA libraries, four library replicates (lib1-4) were prepared for Samples A, B, and C using AGLR1, AGLR2, and ROCR2. * Three library replicates (lib1-3) were prepared using ROCR1. Sample D was sequenced with ROCR1 instead of Sample C. For targeted RNA libraries, four library replicates (lib1-4) were prepared for Samples A, B, C, D, and E using 7 panels. **(d)** Targeted RNA libraries of Sample A, B, and C were made with three panels, each library was split into different fractions (F1, F1 + 2, or F3), and sequenced with both long-read sequencing platforms. **(e)** † ROCR2 was only used for Sample A and the libraries was sequenced only on Nanopore. ‡ Sample B was sequenced by Nanopore Direct RNA protocol only. **(f)** An illustration shows the flexible options for possible comparison analyses for an in-depth study of the impacts of targeting, size selection, and sequencing protocols.

RNA Reference Sample A in this study is identical to the Sample A utilized in the SEQC1 studies^44,48^. DNA Reference Samples A and B were well-characterized in a previous study^48^ under the umbrella of the FDA-led SEQC2 project^1^. Briefly, both RNA Sample A and DNA Sample A were derived from the Agilent Universal Human Reference (UHR) sample^49^, which were pooled from ten cancer cell lines, including brain, breast, liver, B lymphocyte, testis, macrophages, T lymphoblast, liposarcoma, skin, and cervix. The RNA and DNA SEQC2 Sample B was from a cell line derived from a normal male individual (Agilent OneSeq Human Reference DNA, PN 5190–8848). Samples C, D, and E were mixtures of samples A and B in the ratios of 1:1, 1:4, and 4:1 respectively (Fig. 1a). Although DNA Reference Samples A, B, C, and D are defined identically to Samples A, B, C, and D used in previous related studies^45,48^, Sample E is defined as a different admixture in this study. This study included eight oncopanels. The panel codes seen throughout this manuscript are explained in Table 2. Panels abbreviated by “AGLRx” use Agilent, Inc. technology, panels abbreviated as “ROCRx” use Roche, Inc. technology, and panels abbreviated as “ILMRx” use Illumina, Inc. technology. Although AGLR1 and ROCR1 panels were designed for DNA sequencing, and other target sequencing panels were designed for RNA sequencing, some panels were used for both DNA and RNA sequencing regardless of their original design. Targeted DNA libraries were prepared with four panels, AGLR1, AGLR2, ROCR1, ROCR2, and then sequenced with Illumina short-read sequencing. The targeted RNA libraries were prepared with eight panels, AGLR1, AGLR2, ROCR1, ROCR2, ROCR3, ILMR1, ILMR2, ILMR3, and then sequenced with Illumina short-read sequencing except for ROCR3 (Fig. 1b). Targeted RNA libraries for AGLR2, ROCR2, ROCR3 were also sequenced with Nanopore and/or PacBio long-read sequencing, following previously published protocols^21,50^ (Fig. 1b). Whole transcriptome RNA libraries were prepared with four methods: 1) rRNA depletion and 2) poly(A) selection libraries were sequenced with Illumina short-read sequencing, 3) PacBio WTS libraries were sequenced with the PacBio long-read sequencing, and 4) Nanopore Direct RNA libraries were sequenced with Nanopore long-read sequencing (Fig. 1b). Four DNA library replicates were made for Sample A, Sample C (or Sample D for panel ROCR1 instead), and Sample B, for each of the four panels, and four short-read RNA-seq library replicates were made for Samples A, B, C, D, and E, for each of the eight panels (Fig. 1c). Targeted RNA libraries were also made with three panels for Sample A, B, and C, after which each library was split into fractions (F1, F1 + 2, or F3) of different cDNA fragment length distributions. Fractions F1, F1 + 2, and F3 had incrementally greater median fragment lengths. The captured cDNA products were checked on a Bioanalyzer (Agilent Technologies, Santa Clara, CA) to confirm their quality and length distribution. In general, the sequencing reads exhibited read length distributions similar to those measured by Bioanalyzer, and PacBio reads were generally longer than Nanopore reads for the same cDNA products. Fractions F1 and F3 for panels ROCR3 and AGLR2 were sequenced with both Nanopore and PacBio long-read sequencing platforms, while F1 and F1 + 2 for panel ROCR2 were sequenced with the Nanopore long-read sequencing platform only (Fig. 1d). Due to the differences in probe lengths and adjustments to the capture protocols, the fractions captured by AGLR2 were usually shorter than the corresponding fractions captured by ROCR2 and ROCR3. Four library replicates were made for each of rRNA depletion and poly(A) selection methods for Samples A, B, and C. One library for Sample A was made and sequenced with both PacBio WTS and Nanopore Direct RNA methods, and one library for Sample B was made and sequenced with Nanopore Direct RNA method (Fig. 1e). In addition, RNA libraries of the ten Agilent UHR cell lines were captured using a ROCR3 panel and sequenced on Nanopore and PacBio sequencing platforms.

This comprehensive experimental design includes built-in known information through dilution sequences and allows an interrogation of the effects of the different short- and long-read sequencing technologies, targeting panels, library preparation methods, and fragment size selection options (Fig. 1f). The experimental design for the reference benchmark study is shown in Table 1. As part of the SEQC2 study, we have published a comprehensive study detailing the creation of reference Samples A and B, along with the (variant) positives and negatives within our regions of interest, specifically the consensus target region (CTR)^48^. Additionally, we identified and reported an extended set of indels within the CTR and the exon regions of the Catalogue of Somatic Mutations in Cancer (COSMIC) Cancer Gene Census through an extensive manual review^51^. These positives, indels, and negatives can be utilized to benchmark variant calling pipelines, as demonstrated in a community indel calling challenge hosted on the precisionFDA platform^52^. Furthermore, we released three whole-exome sequencing datasets for Samples A and B in our published study^48^.

**Table 1:** Experimental design and data availability.

		Reference Samples										
		A			E	C			D	B		
		SR*	LR*		SR	SR	LR		SR	SR	LR	
		I*	N*	P*	I	I	N	P	I	I	N	P
**Targeted RNA-seq**	AGLR1	4^†^			4	4			4	4		
	AGLR2	4	F1, F3^‡^	F1, F3	4	4	F1, F3	F1, F3	4	4	F1, F3	F1, F3
	ROCR1	4			4	4			4	4		
	ROCR2	4	12^§^		4	4			4	4		
	ROCR3		F1, F3	F1, F3			F1, F3	F1, F3			F1, F3	F1, F3
	ILMR1	4			4	4			4	4		
	ILMR2	4			4	4			4	4		
	ILMR3	4			4	4			4	4		
**Targeted DNA-seq**	AGLR1	4				4				4		
	AGLR2	4				4				4		
	ROCR1	3							3	3		
	ROCR2	4				4				4		
**Total RNA-seq**	rRNA depletion	4				4				4		
	Poly(A) selection	4				4				4		
	PacBio WTS		1									
	Nanopore Direct RNA		1							1		

* SR stands for short-read sequencing, LR stands for long-read sequencing, “I” stands for Illumina platform, “N” stands for Nanopore platform, “P” stands for PacBio platform.

^**† **^The numbers 1, 3, 4 are the numbers of technical replicates.

^**‡ **^F1, F2, and F3 are the two fragment selection methods.

^**§ **^The 12 replicates arise from the combination of two capture methods (single and double capture), two fragment selection methods (F1 and F1 + 2 combining F1 and F2), and three technical replicates.

### Characteristics of examined panels and sequencing data

Three oncopanel providers joined this study and contributed a total of eight oncopanels for target capture. We distributed reference samples to the laboratories, where a combined total of 430 cDNA libraries were prepared. Detailed information of the eight participating oncopanels are listed in Table 2. To shorten the description and file names, panel codes were used to identify panels. We mapped the probe sequences to the reference genome and transcriptome using the Magic pipeline^53^. Probes for some panels were originally designed for genomic sequence, others for transcriptome sequences, while others used a hybrid approach (see Experimental protocols). Although most probes aligned over their entire lengths on the genome or the transcriptome, we also accepted mappings with at least 80% aligned probe length. Table 2 shows the size of the genomic sequence and the number of RefSeq genes which were targeted by the probes. Statistics for other gene model annotations can be found in the Supplement.

**Table 2:** Characteristics of targeted panels examined.

Panel Code	Panel Name*	hg19/GRCh37		Hg38/GRCh38	
		Mapping size on genome* (Mbp)	Number of RefSeq genes targeted^†^	Mapping size on genome* (Mbp)	Number of RefSeq genes targeted^†^
AGLR1	Agilent Clear-seq custom comprehensive cancer DNA panel	7.67	1113	7.94	1163
AGLR2	Agilent custom union panel	16.37	2225	17.00	2277
ILMR1	Illumina TruSight^TM^ Tumor 170 RNA panel	0.40	56	0.40	57
ILMR2	Illumina RNA fusion panel	NA^‡^			
ILMR3	Illumina whole exome RNA panel	31.01	20448	32.18	20822
ROCR1	Roche comprehensive cancer DNA	2.95	1005	3.05	1034
ROCR2	Roche custom union panel	17.09	2322	17.79	2377
ROCR3	Prioritized subset of Roche custom union panel	4.71	629	4.74	644

* The mapping size on genome was calculated by the mega base-pairs (Mbp) of the reference genome covered by the probes. The mapping of the probes to the reference genome was done using Magic pipeline.

^**† **^The gene count is the number of RefSeq genes (v105 for hg19, v109 for hg38) which are targeted by the probes.

^**‡ **^The ILMR2 panel is specifically designed for targeting fusion junction, thus is not applicable to calculate the numbers in this table.

For targeted panels, the total read pairs from short-read sequencing yielded about 38 M per RNA library replicate in average, and 125 Mbps per DNA library replicate; while the total reads from long-read sequencing yielded about 5.4 M per RNA library replicate in average. The sequencing quality was good for the short-read sequencing, where the quality score of more than 95% reads was no less than 30. Sequencing quality the Nanopore and PacBio long-read sequencing was generated and analyzed separately. Detailed information can be found in Supplemental Table 1.

### Experimental protocols

#### Reference sample RNA and DNA library construction

As part of the FDA-led SEQC2 project, the description of the reference samples, the preparation of the DNA and RNA libraries, and the sequencing protocols contains overlap with our previous SEQC^41^ and SEQC2^44,54–56^ publications due to the standardized and well-established nature of the NGS procedures. Here, we provided the specific details pertinent to this study. The RNA samples utilized in this study were kindly prepared and provided by Agilent Technologies. Sample A here was the well characterized and widely used Universal Human Reference RNA (UHRR, from ten pooled cancer cell lines of equal mass, Agilent Technologies, Inc.)^49^. To complement Sample A, we introduced a new RNA reference Sample B, created by extracting total RNA from a normal cell line (Agilent Human Reference DNA, Male, Agilent part #: 5190–8848). Samples A and B were then combined in ratios of 1:1, 1:4, 4:1 respectively, to generate samples C, D, and E. Total RNA from each UHRR cell line was provided. All Samples had high quality, with a RIN above 9.2 and a DV200 above 92%. Samples A, B, C, D, and E were aliquoted at 5 μg per 1.5 ml tube at 200 ng/μL concentration. It is worth pointing out the differences between these samples and reference samples used in the previous SEQC1 projects^41,57^, where Sample B was a Human Brain Reference RNA sample. Matching the sample design in our SEQC2 oncology panel sequencing study^44,48^, we feature a new RNA reference Sample B. The names and mixing ratios for Samples C and D are also identical across the studies. However, the Sample E in this paper had a mixing ratio different to that of the DNA reference Sample E in the SEQC2 reference sample and liquid biopsy study^48,54,55^. A choice of symmetric mixing ratios for Samples D and E in this study is better suited to an evaluation of gene expression quantification.

To match the reference RNA samples, DNA Sample A was composed of a near equal mass pooling of 10 gDNA samples prepared from Agilent’s UHRR cancer cell lines. Sample B was a gDNA sample from the normal male cell line (Agilent Human Reference DNA, Male, Agilent part #: 5190–8848). Samples C and D were a 1:1 and 1:4 mix of Samples A and B, respectively. Samples A, B, C, and D were aliquoted at 3 μg per 1.5 ml tube in low-EDTA TE buffer (10 mM Tris, 0.1 mM EDTA, pH 8.0) at 20 ng/μL concentration.

#### Targeted Short-read RNA-seq

Eight targeted sequencing panels were used for the short-read RNA-seq. The basic information of these panels, including the panel name, mapping size on genome, and the number of targeted genes can be found in Table 2. All eight panels use hybrid capture-based target enrichment as its capture method.

##### AGLR1 and AGLR2 targeted RNA-seq for RNA Samples A, B, C, D, and E

The detailed protocol for Agilent targeted RNA-Seq “*SureSelect*^*XT*^ *RNA Direct for Preparation of Strand-Specific Sequencing Libraries from High-Quality or FFPE-Derived RNA Samples for the Illumina Platform*” (part number G9691-90050) and can be accessed with the following link: https://www.agilent.com/cs/library/usermanuals/public/G9691-90050.pdf.

Five different total RNA samples (samples A, B, C, D, and E) were provided at a concentration of 200 ng/μL. These samples were diluted to 50 ng/μL and the concentrations were verified by quadruplicate Nanodrop measurements for each of the 5 different total RNA samples. Based on the Nanodrop concentrations, quadruplicate reactions with 100 ng total RNA input were set up for each of the RNA samples. The RNA was lyophilized to dryness at medium heat in a Speed-vac and resuspended in fragmentation buffer. The total RNA samples were chemically fragmented at 94 °C for 8 minutes then cooled to 4 °C. The fragmentation mix contains the primers necessary for cDNA conversion which are annealed during the fragmentation step.

To maintain strand-specificity, fresh Actinomycin D was prepared and added to the first strand master mix. This master mix was added directly to the fragmented RNA sample and the first strand reaction was incubated at 25 °C for 10 minutes followed by a 37 °C incubation for 40 minutes. The samples were purified with AMPure XP beads and the second strand master mix containing end-repair reagents was added to the eluted samples followed by an incubation at 16 °C for one hour. The samples were purified using AMPure XP beads where the resulting cDNA was A-tailed at 37 °C for 30 minutes followed by the addition of adapter ligation mix and incubation at 20 °C for 15 minutes. The cDNA was purified again using AMPure XP beads and the eluted cDNA was treated with uracil DNA glycosylase at 37 °C for 15 minutes followed by 14 cycles of PCR amplification. Pre-capture PCR yields and cDNA fragment sizes were measured using a 2200 TapeStation High Sensitivity D1000 assay (TapeStation D1000).

Based on the TapeStation D1000 pre-capture concentrations, 200 ng cDNA for each sample was prepared for targeted hybridization by first annealing blocker oligos at 95 °C for 5 minutes and then samples were maintained at 65 °C for the hybridization. Biotinylated 120-mer oligos corresponding to the either the Agilent AGLR1 panel or the AGLR2 panel were added to capture transcripts of interest in an overnight hybridization of 24 hours at 65 °C. Dynabeads M270 streptavidin beads were used to capture the hybridized cDNA libraries. After three rounds of washing the cDNA libraries were not eluted from the M270 beads, and instead half of each of the resuspended bead mixture was PCR amplified using primers containing unique 8 bp (base pair) molecular indexes to uniquely mark each technical replicate sample. After 12 cycles of post-capture PCR AMPure XP beads were added and the final cDNA libraries were eluted. Final library concentrations and fragment sizes were determined using a TapeStation HSD1000 tape. Based on the molar concentrations for each of the four replicates for each of the 5 RNA samples the 20 uniquely indexed samples for each of the two Agilent panels were pooled in equimolar concentrations to a final concentration of 10 nM and sequenced on an Illumina HiSeq® 2500.

Agilent prepared two different RNA panels in the SEQC2 project: AGLR1 and AGLR2. AGLR1 was the same panel used in our previous study^44^. AGLR2 was specially designed in the consortium to create a comprehensive unified research onco-panel. This panel was well suited for assessing alternative splicing because these genes are known to feature complex splicing variants. Briefly, we targeted genes from established onco-panels and additional genes of interest, including FDA approved cancer biomarkers, ACMG genes^58,59^ recommended for reports of secondary findings, HLAs, DMETs, genes repeatedly observed in fusions in breast cancer, and other cancer related genes. This resulted in 2,125 unique AceView genes^60^ (Supplemental Fig. 1). Considering a typical Illumina fragmentation length of 180–210 the resulting spacer length of <60 bases ensured that capture was uniform across the entire transcript lengths. To make the panel suitable for DNA as well as RNA capture, we avoid probes spanning exon-exon junctions where possible. For exons shorter than 120 bases, however, we design capture probes for *all* the known exon junctions, prioritizing RNA capture by design. About 12% of probes span exon-exon junctions though, yielding a panel highly efficient for both targeted RNA-Seq and DNA-Seq.

The pre-capture yields were high enough to perform the capture hybridization steps with the two different Agilent panels using the same pre-capture cDNA libraries. The smaller AGLR1 panel was run on four different lanes of an HiSeq® 2500 and the larger AGLR2 panel was run on 5 lanes of an HiSeq® 2500, generating approximately 50 million paired reads per indexed sample/panel.

##### ROCR1 and ROCR2 targeted RNA-seq for Samples A, B, C, D, and E

The ROCR1 panel content was based on a list of 1048 genes involved in either hereditary oncology or somatic oncology. Coding regions from over 6350 transcripts were extracted from CCDS, RefSeq and Ensembl annotations sources and used to define a set of 16,146 genomic regions in hg38, totaling 2.75 Mbp. Candidate probes (min 50 bp; max 100 bp; avg 75 bp) were generated at a 5 bp interval for the entire sequence set. Probes were screened for repetitiveness by calculating the average frequency of each 15-mer in the probe sequences. Probes with a value of 100 were discarded. Probe sequences were then converted to FASTA and compared to the genome using SSAHA (v1), with a close match being defined as a minimum match size of 30 bp and < = 5 mismatches/insertions/deletions. Probe positions and *in silico* metrics (homopolymer composition, number of matches in the genome, repetitiveness score) were then loaded into a MySQL table. Capture probes were selected for each coding sequence feature by scoring one to three probes in a 15-base window, based on repetitiveness, uniqueness, melting temperature, and sequence composition, and then choosing the best capture probe in that window. The start of the 20-base window was then moved 35 bases downstream and the process repeated. This resulted in an average probe spacing of approximately 35 bp. This panel was originally designed for DNA capture; probes were selected from the top strand of the genome, and the manufactured probes were complementary to that top strand. The final panel consisted of 64,343 unique probes, with a total consolidated size of 2.93 Mbp. Probe sequences were supplied to the Working Group, for alignment to both hg19 and hg38 genome builds, and transcript annotation.

The ROCR2 panel’s starting point was the same set of genes targeted for AGLR2 plus six additional genes of fusion interest (ARFGEF2, NPEPPS, RASA3, SULF2, TBC1D3, TMEM49), for a total of 2,131 panel genes with 27,737 AceView^60^ transcript sequences. Candidate probes were generated as described above, with the exception that the repetitive score threshold was raised to 1000. Sequence redundancy in the transcript set was removed by looking for the first instance of each distinct 50-mer sequence and then masking subsequent occurrences of that 50-mer in the transcript set. This left a non-redundant set of targets for probe selection, where individual exons were generally covered once in the exemplar transcript, and the set of exon-exon junctions was covered for every unique combination. Probes were tiled across the unique regions as described above, and the probes were designed in such a way that the final biotinylated capture probes would capture the sense strand of the transcript, allowing both direct RNA capture as well as cDNA capture. The final panel consisted of 449,690 unique probes and a total consolidated size of 48.52 Mbp. Probe sequences were supplied to the Working Group, for alignment to both hg19 and hg38 genome builds and transcript annotation. Targeted RNA sequencing libraries of samples A, B, C, D, and E, provided by Agilent (Agilent Technologies), were prepared in accordance with Roche’s SeqCap RNA Enrichment System User’s Guide (version 1.1). In brief, 100 ng of each RNA sample with four technical replicates was pooled with 2 µL of 1:1000 diluted ERCC RNA Spike-In Control mix 1 (Life Technologies). RNA libraries were first constructed using KAPA Stranded RNA-Seq Library Preparation kit with 11 cycles of pre-capture PCR amplification, and 1 µg of each amplified library was then individually hybridized with 4.5 μL of Roche probe pools, ROCR1 or ROCR2, at 47 °C for 20 hours. After hybridization, the probe-target complexes were captured with streptavidin-coated SeqCap Pure Capture Beads, and then washed sequentially with wash buffers to remove non-targeted products. Captured libraries were further amplified by 14 cycles of PCR. Targeted RNA sequencing libraries from each probe pool were mixed in equimolar amounts and sequenced on an Illumina HiSeq® 2500.

##### ILMR1 targeted RNA-seq for Samples A, B, C, D, and E

RNA samples were processed according to the TruSight^TM^ Tumor 170 Reference Guide (https://support.illumina.com/content/dam/illumina-support/documents/documentation/chemistry_documentation/trusight/tumor-170/trusight-tumor-170-reference-guide-1000000024091-02.pdf). Briefly, cDNA was generated for each sample, followed by a SPRI clean-up. End-repair, adapter ligation, post-ligation clean-up, indexing, and target capture was performed as previously described^56^. Target specific oligos which cover 357 kb of genomic targets across 55 genes, followed by capture with streptavidin magnetic beads. A second hybridization, capture, PCR amplification, library normalization, pooling, and sequencing was performed as described previously^56^.

##### ILMR2 targeted RNA-seq for Samples A, B, C, D, and E

Libraries were prepared using the TruSight^TM^ RNA Pan-Cancer Panel Reference Guide. Briefly, cDNA was generated from RNA followed by a SPRI clean up. cDNA was A-tailed, index ligated, cleaned-up and PCR amplified as previously described^56^. Target regions were captured using a 90-minute hybridization to biotinylated target specific oligos covering 533 kb of genomic targets across 1,385 genes, followed by capture with streptavidin magnetic beads. Second hybridization, capture, and PCR amplification was performed as previously described^56^. Libraries were quantified and manually normalized to 6 nM before being pooled in equal parts per library. Libraries were then further diluted and loaded, 20 libraries per Illumina NextSeq^TM^ v2 high-output flowcell. Sequencing was performed as 2 × 101 bp with 6 bp single indexed reads.

##### ILMR3 targeted RNA-seq for Samples A, B, C, D, and E

Libraries were prepared using the TruSeq^TM^ RNA Exome Reference Guide. Briefly, cDNA was generated from RNA followed by a SPRI clean up. cDNA was then A-tailed, followed by ligation to a uniquely indexed adapter. Post-ligation clean-up was performed using SPRI beads and then libraries were PCR amplified. Target regions were captured using a 90-minute hybridization to biotinylated target specific oligos covering 45.3 Mb of genomic targets across 21,415 genes, followed by capture with streptavidin magnetic beads. A second hybridization and capture reaction was performed followed by PCR amplification using the universal primers compatible with the sequencing flowcell. Libraries were quantified and manually normalized to 6 nM before being pooled into two 10-plex pools in equal parts per library. Libraries were then further diluted and sequenced on an Illumina NextSeq^TM^ v2 high-output flowcell. Sequencing was performed as 2 × 101 bp with 6 bp single indexed reads.

#### Targeted Short-read DNA-seq

##### AGLR1 targeted DNA-seq for DNA Samples A, B, and C

The AGLR1 targeted DNA-seq data was borrowed from our previous SEQC2 study^44^. Genomic DNA libraries were constructed for the test samples according to the Agilent SureSelectXT HS Target Enrichment System for Illumina Paired-End Multiplexed Sequencing Library Protocol (Cat. No. G9702-90000 Version A1, July 2017). In brief, 30 ng of each cell line’s high molecular weight genomic DNA was sonicated in a 50 μL volume, using a Covaris E220 instrument to a mean size of 350 bp (Duty Factor: 10%, Peak Incident Power: 175, Cycles per Burst: 200, Treatment Time: 2 × 30 seconds, Bath Temperature: 2° to 8 °C). DNA fragments were then end-repaired and A-tailed using a two-step cycling protocol (20 °C for 15 minutes and 72 °C for 15 minutes), followed by ligation to XTHS adaptors with UMIs for 30 minutes at 20 °C. Adapter-ligated fragments were amplified and indexed by PCR in a 50 μL total volume with Herculase II Fusion DNA Polymerase under the following conditions: 2 min at 98 °C (initial denaturation), 10 cycle amplification of 30 seconds at 98 °C, 30 seconds at 60 °C, 1 minute at 72 °C, and 5 minutes at 72 °C (final extension). Library quality control (quantity and size distribution) was then assessed using either the 2100 Bioanalyzer High Sensitivity DNA 1000 assay (Bioanalyzer 1000) or the TapeStation D1000. 1 μg of prepared gDNA libraries were then hybridized to a custom Immuno-Oncology focused Comprehensive Cancer Panel (1,058 targets coding regions including UTRs and 7.6 Mb in size) biotinylated RNA probes (5 minutes at 95 °C, 10 minutes at 65 °C, 1 minute at 65 °C, 60 cycles of 1 minute at 65 °C and 3 seconds at 37 °C, and 65 °C hold) and captured with Dynabeads MyOne Streptavidin T1 beads. SureSelect enriched gDNA libraries were PCR amplified using an on-bead protocol in a 50 μL volume with Herculase II Fusion DNA Polymerase under the following conditions: 2 min at 98 °C (initial denaturation), 10 cycles of 30 seconds at 98 °C, 30 seconds at 60 °C, 1 minute at 72 °C (amplification), and 5 minutes at 72 °C (final extension), followed by 4 °C hold. All DNA purifications between steps were performed using AMPure XP beads as indicated in the user manual. Post-capture library quality control was again assessed using either the Bioanalyzer 1000 or the TapeStation D1000. Indexed samples were finally pooled and sequenced to approximately 5,000X (Samples A, B) or 10,000X (Sample C) read depth on a NovaSeq^TM^ 6000 instrument using a 2 × 150 bp paired-end protocol (Q30 scores ≥ 75%).

##### ROCR2 and AGLR2 targeted DNA-seq for DNA Samples A, B, and C

Genomic DNA samples A, B, and C were provided by Agilent (Agilent Technologies). Targeted DNA sequencing libraries were constructed according to Roche’s SeqCap EZ HyperCap Workflow User’s Guide (version 1.2), or Agilent’s protocol of SureSelectXT Target Enrichment System for Illumina Paired-End Multiplexed Sequencing Library (version C3). In brief, genomic DNA samples were sonicated and sheared to approximately 200 bp fragments using a Covaris S220 System. 100 ng of each fragmented DNA sample in four technical replicates was used the input for library preparation. The samples were sequentially end‐repaired, A‐tailed and adapter‐ligated. The ligated products were then subjected to minimal PCR cycling as suggested by the protocol and quantified with Agilent high sensitivity DNA 1000 assay (Agilent 1000 assay). Amplified libraries were individually hybridized overnight with Roche ROCR2 panel, or Agilent AGLR2 panel, respectively. The hybridized libraries were captured with streptavidin-coated beads and washed sequentially with wash buffers. Captured libraries were further amplified with 14 cycles of PCR, and the quality of the libraries was validated by the Agilent 1000 assay. The libraries from each panel were pooled in equimolar amounts and subjected to 150 bp paired-end sequencing (PE150) on an Illumina NovaSeq^TM^ system.

##### ROCR1 targeted DNA-seq for DNA Samples A, B, and D

Genomic DNA samples A, D, B, were provided by Agilent (Agilent Technologies). Targeted DNA sequencing libraries were constructed using KAPA Hyper Prep kit (Kapa Biosystems), and Roche NimbleGen SeqCap EZ hybridization and wash kit (Roche NimbleGen Inc) as per Roche SeqCap EZ HyperCap Workflow User’s Guide (version 1.2). In brief, genomic DNA samples were sonicated to achieve a mode fragment length of 200 bp on a Covaris S220 System in a 50 µl volume according to the manufacturer’s specifications. 100 ng of each fragmented DNA sample in triplicates was used for library preparation. The samples were sequentially end‐repaired, A‐tailed and adapter‐ligated. After double-sided size selection with Agencourt AmPure XP beads, the resulting libraries were subjected to 9 cycles of PCR amplification and quantified with the Agilent 1000 assay. 1 μg of each library was individually hybridized with 4.5 μL of Roche ROCR1 probes at 47 °C for 20 hours. After incubation with streptavidin-coated SeqCap Pure Capture Beads at 47 °C for 15 minutes, the libraries were washed sequentially with wash buffers to remove non-targeted products. The enriched libraries were further amplified by PCR with 14 cycles. Final libraries were validated by Agilent’s 1000 assay and quantitative PCR. The libraries were pooled in equimolar amounts and subjected to 100 bp paired-end sequencing on an Illumina HiSeq® 2500.

#### Targeted Long-read RNA-seq

##### PacBio sequencing of long cDNA captured by ROCR3, ROCR2, and AGLR2

The ROCR3 panel is a subset of the ROCR2 panel, targeting a prioritized selection of 580 genes (based on AceView gene model) from that panel. The same probe sequences were utilized, filtering based on gene/transcript name. The final panel consisted of 141,630 unique probes and a total consolidated size of 14.34 Mbps. Probe sequences were supplied to the Working Group, for alignment to both hg19 and hg38 genome builds and transcript annotation.

The prioritized gene set has been selected for higher efficiency capture and by interest to the community, so that the potential of long reads can be exploited despite the lower sequencing depths often obtained from long read technologies (see Supplement for details). Except for ARFGEF2 and RASA3, these genes were also on the AGLR2 panel.

For ranking, we prioritized target genes:

- on established panels or in gene sets of interest to the community,
- with newly predicted fusions,
- with a non-trivial (complex) exon structure but not singular (unsolvable),
- with differentially expressed transcripts when the gene is not differentially expressed,
- where transcripts and the gene are differentially expressed in opposite directions, or
- where different transcripts are differentially expressed in opposite directions.

This was done while avoiding genes with extremely high expression or with a single transcript dominating expression at all times. The somewhat arbitrary score functions have been designed to identify multi-modal distributions (such as arising from ‘something’ *vs* ‘nothing’) and, taking the underlying score distributions into account, to have an effect on a reasonable proportion of candidates. Extreme expression Z-scores contribute the most to the sorting, as large expression of individual targets can negatively affect the whole panel and are thus punished aggressively.

All total RNA samples were provided by Agilent (Agilent Technologies). Full-length cDNA preparation with size selection for Sequel Systems was carried out by following the PacBio Iso-seq protocol (PN 101-070-200 Version 05). Briefly, 1 μg of total RNA from each sample as indicated was used as input for cDNA synthesis reactions (three or more as needed) using a Clontech SMARTer PCR cDNA Synthesis kit. After PCR cycle optimization, a total of 11 cycles of PCR amplification were adapted to generate the large-scale double-strand cDNA using the Takara PrimeSTAR GXL DNA Polymerase kit. The PCR products were pooled together, and split into different fractions: Fraction 1 (F1) and Fraction 2 (F2) were purified with 1 × or 0.4 × PacBio AMPure PB beads, respectively. Fraction 3 (F3) was purified with 1 × AMPure PB beads, followed by > 4 kb size selection using Sage Science BluePippin Size Selection System as described in the protocol. The post-size selection products were further amplified by PCR, using 6 cycles and re-purified with 0.5 × AMPure PB beads.

Hybridization was processed according to the instructions of PacBio cDNA capture using SeqCap® EZ Libraries (PN 101-601-200 Version 01) with some adaptations. 1.5 μg of the cDNA fractions, F1, F2, and F3, as well as F1 + 2 (an equimolar mixture of F1 and F2), were individually hybridized overnight with the capture panels of ROCR2 and ROCR3 from Roche at 47 °C, or AGLR2 from Agilent at 65 °C. The hybridized products were incubated with Roche SeqCap Pure Capture Beads for ROCR2 and ROCR3, or Invitrogen Dynabeads M-270 for AGLR2, and then washed sequentially with wash buffers. For double capture, the post-hybridization F1 and F1 + 2 fractions of Sample A from single capture with ROC2 were amplified by PCR with 5 cycles and purified with 1 × AMPure PB beads. The amplified products were re-hybridized overnight with ROC2 at 47 °C. The captured fractions were amplified by PCR with PrimeSTAR GXL DNA Polymerase kit for ROCR2 and ROCR3, or KAPA HiFi HotStart ReadyMix PCR kit for AGLR2. The resulting cDNA samples were evaluated and quantified using the Agilent DNA 12000 assay and the Qubit dsDNA High Sensitivity assay, respectively, and subjected to Oxford Nanopore sequencing (see details below) and/or PacBio sequencing.

Three sets of captured cDNA samples were created. Set 1 consisted of fractions F1 and F3 for all 10 cell line samples, Sample A, Sample B captured by ROCR3. Each sample from Set 1 was sequenced by both long read sequencing technologies. More specifically, after construction of Single Molecule Real Time (SMRT) bell libraries, each sample was sequenced in one SMRT Cell 1 M on a PacBio Sequel instrument. Set 2 consisted of fractions F1 and F1 + 2 from Sample A captured by ROCR2 with three replicates. Samples in Set 2 were sequenced by Nanopore only. Set 3 consisted of fractions F1 and F3 from samples A, C, B captured by ROCR3 or AGLR2. In total, there were 12 captured cDNA samples in Set 3. Each sample from Set 3 was sequenced by both technologies. Each sample was run on one SMRT Cell 8 M was used for each sample on a PacBio Sequel II system.

##### Nanopore sequencing of Sample A cDNA samples single and double captured by ROCR2

12 cDNA libraries were sequenced on an ONT PromethION. Briefly, 200 fmol of cDNA was taken into a genomic DNA by ligation (SQK-LSK109) prep from ONT. Libraries were barcoded with ONT’s native barcoding kit (EXP-NBD103) – 2 combined library pools were created (6 samples each, see supplement) with special attention to fragment size to avoid sequencing bias. These were run on the PromethION Beta sequencing device using a FLO-PRO002 flowcell and run for 64 hours. FASTQ files were generated with Guppy Basecaller 3.6.1 (https://github.com/nanoporetech/pyguppyclient).

##### Nanopore sequencing of cDNA samples A, B, and C captured by ROCR3

6 cDNA libraries were run on an ONT PromethION. Briefly, 200 fmol of cDNA was taken into a genomic DNA by ligation (SQK-LSK109) prep from ONT. Libraries were barcoded with ONT’s native barcoding kit (EXP-NBD104) – 2 combined library pools were created (3 samples each, see supplement) with special attention to fragment size to avoid sequencing bias. These were sequenced on the PromethION Beta sequencing device using a FLO-PRO002 flowcell and run for 64 hours. FASTQ files were generated with Guppy Basecaller 3.6.1 (https://github.com/nanoporetech/pyguppyclient).

##### Nanopore sequencing of cDNA samples A, B, and C captured by AGLR2

6 cDNA libraries were run on an ONT PromethION. Briefly, 200 fmol of cDNA was taken into a genomic DNA by ligation (SQK-LSK109) prep from ONT. Libraries were barcoded with ONT’s native barcoding kit (EXP-NBD104) – 2 combined library pools were created (3 samples each) with special attention to fragment size to avoid sequencing bias. These were sequenced on the PromethION Beta sequencing device using a FLO-PRO002 flowcell and run for 64 hours. FASTQ files were generated with Guppy Basecaller 3.6.1 (https://github.com/nanoporetech/pyguppyclient).

#### Whole transcriptome RNA-seq

##### Whole Transcriptome RNA-seq of RNA Samples A, B, and C

RNA Sample A, C, B were sent to HudsonAlpha Discovery Life Sciences (DLS, https://gslweb.discoveryls.com/index) for library preparation and deep sequencing. Briefly, each of the three samples was prepared with two library preparation methods: strand-specific poly(A) selection and strand-specific ribosomal depletion. Each preparation type was replicated four times for each sample, producing a total of 8 libraries from each of the three samples. RIN values were determined with Bioanalyzer 1000 prior to library preparation. Sample A had a RIN of 8.8 and DV200 of 92%, sample B had a RIN of 9.5 and DV200 of 95% and sample C had a RIN of 9.2 and DV200 of 93%. A total of 250 ng of total RNA was used input into each reaction. Poly(A) selected library preparation was performed using the NEB Ultra II kit (New England Biolabs) and rRNA reduction library preparation was performed with Illumina Stranded Total RNA Prep Ligation with Ribo-Zero^TM^ Plus (Illumina). Both protocols were performed per manufacturer’s direction with the substitution of Illumina standard paired-end adapters (Integrated DNA Technologies) used at the ligation step and unique-dual indexing primers (Integrated DNA Technologies) added at PCR in both protocols. After library generation, quantification was performed by PicoGreen (ThermoFisher Scientific), library sizing was performed by Caliper fragment analysis (PerkinElmer), and qRT-PCR quantitation using the Roche/Kapa library quantification kit. Successful libraries yielded approximately 500 ng final library with insert sizes in the 350–500 bp range based on fragment analysis. All libraries passed the above quality metrics. Libraries were normalized to 1.4 nM concentration based on Kapa qPCR results and pooled in equal amounts for sequencing on an Illumina NovaSeq^TM^ 6000 instrument using v1.0 sequencing reagents on the S4 flowcell at PE150 conditions. Each sample was sequenced to greater than 100 M paired reads per sample. FASTQ files were demultiplexed and transferred to the data repository at NCBI.

##### Whole Transcriptome Sequencing of Sample A total RNA by PacBio

Whole transcriptome RNA libraries of Sample A were prepared and sequenced by Pacific Biosciences. In brief, cDNA was prepared from Sample A total RNA using the Clontech SMARTer PCR cDNA Synthesis Kit, where poly(dT) primers targeted full length transcripts with a poly(A) tail. The libraries were then cleaned using AMPure beads and we performed a QC prior to setting up a sequencing run. The sequencing library was prepared with the Iso-Seq Template Preparation for Sequel Systems (PN 101–070-200) and Sequencing Sequel System II with “Early Access” binding kit (101–490-800) and chemistry (101–490-900). The sequencing library was sequenced on eight Sequel II SMRT cells of 15 hours run time per SMRT Cell. The sequencing data was processed into CCS reads using the ccs tool with the parameters “–noPolish–minPasses = 1”. CCS reads with cDNA primers and poly(A) tails were identified as full-length, non-concatemer (FLNC) reads using lima (–isoseq–dump-clips) and isoseq. 3 refine (–require-polya).

##### Direct RNA Sequencing of Samples A and B by Oxford Nanopore

Total RNA of Sample A was run 3 times through the Oxford Nanopore Technologies (ONT) Direct RNA protocol (SQK-RNA002). Briefly, 10 μg of total RNA was used for the library preparation. Poly(A)-tailed RNA is recommended for input, but the library preparation naturally selects poly(A)-tailed RNA and rRNA should be washed away in the bead steps. This approach was chosen to limit manipulation of RNA and maintain quality. After library preparation, library was loaded onto a MinION flowcell (FLO-MIN106D, preferred over FLO-MIN107 for RNA applications) and run for 48 hours. Fastq files were generated with Guppy Basecaller 3.6.1 (https://github.com/nanoporetech/pyguppyclient). This process was repeated 3 times. The Direct RNA experiment was performed once for RNA Sample B.

##### Master table of probe mapping to genes for targeted sequencing panels

Probes of targeted sequencing panels, including AGLR1, AGLR2, ROCR1, ROCR2, ROCR3, ILMR1, ILMR2, and ILMR3, were mapped to gene regions defined by GENCODE (release 36) to identify the gene sets that are covered by each targeted sequencing panel. The mapping result is summarized in Supplemental Table 2. In total, 26,892 genes were targeted by at least one probe of the eight targeted sequencing panels, where 24,113 genes were labeled as “well covered” with MAGIC pipeline.

## Data Records

The data have been deposited to NCBI SRA with accession number SRP437076^61^. There are 240 NCBI SRA records in total for this study. A detailed list of the NCBI SRA records can be found in Supplemental Table 1. The “Library replicate ID” column shows the individual library replicate identifier, which combines the sample ID, panel code, sequencing platform, and library replicate together with “-”. The file names are listed in in columns “filename1” to “filename10”. These filenames are the original filenames used when uploading the data files to NCBI SRA. For the paired-end FASTQ files, “R1” and “R2” are used to indicate the left and right reads. We split bigger files into parts. For some records, there may be “part1” to “part5” in the filenames, which indicates different parts of the same data files.

## Technical Validation

All the data has passed both internal wet-lab and dry-lab quality control to ensure data quality. The average sequencing quality score (Phred score) is from 33 to 37, and the percentage of high-quality reads (Phred > = 30) is above 95% on average for the Illumina platform (Supplemental Table 1). As part of the FDA-led SEQC2 project, this comprehensive study design enabled comparison and cross validation among: (a) sequencing technologies including short-read and long-read sequencing; (b) sequencing platforms including Illumina, PacBio and Nanopore sequencing platforms; (c) targeting regions, defined by seven targeted panels as well as whole transcriptome; (d) DNA and RNA libraries of the same reference samples; (e) samples diluted in different ratios; and (f) technical library replicates that were sequenced using the same protocol that can inform future validation. The previously described SEQC2 study also provided a high confidence list of single nucleotide variants (SNVs) and small indels, as well as known negative positions^48^.

## Usage Notes

The data was supplied in either FASTQ or unmapped BAM format. Short-read sequencing data is paired-end, and long-read sequencing data is single-end. If a library replicate has multiple parts, all parts need to be merged before data processing and analysis. Sequence data files can be downloaded using SRA Toolkit. The original file names are listed in Supplemental Table 1. The AGLR1 targeted DNA-seq data was borrowed from our previous SEQC2 study^44^, in which the panel ID is “AGL”, and the data can be found in the published data descriptor^56^. When interpreting reads, we recommend use of the compiled Master probe mapping table (Supplementary table 2, one sheet per annotation: RefSeq, Gencode and AceView, on hg19/GRCh37 or hg38/GRCh38). The targeted regions of each target panels (provided as BED files) can be downloaded from figshare^62^.

The Agilent RNA reference sample A is a current product, the Agilent DNA reference sample A, and the Agilent RNA and DNA reference samples B are potential products of Agilent Technologies, Inc.

### Supplementary information

Supplemental Figure 1

Supplemental Table 1

Supplemental Table 2

## Data Availability

The data was provided in either FASTQ or unmapped BAM format, generated according to the manufacturers’ experimental protocols as detailed in the Methods section. No custom code was developed for data processing. All software and pipelines utilized for data generation are described in the Methods section. Default settings were used where specific parameters were not specified.
