# Supplementary material for: Targeted DNA-seq and RNA-seq of Reference Samples with Short-read and Long-read Sequencing
Source: Sci Data. 2024 Aug 16;11:892. doi: 10.1038/s41597-024-03741-y (PMC11329654; doi:10.1038/s41597-024-03741-y)
Supplement: Supplementary file 1 — Supplemental Figure 1 [file 41597_2024_3741_MOESM1_ESM.docx]

**a**

on.Qiagen on.Thermo on.xGen on.ill170 on.ill1k on.agi151 on.agi1k on.roche

275 141 127 170 1378 151 1043 998

**
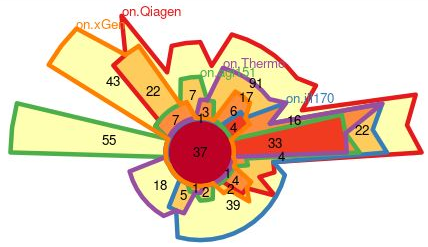

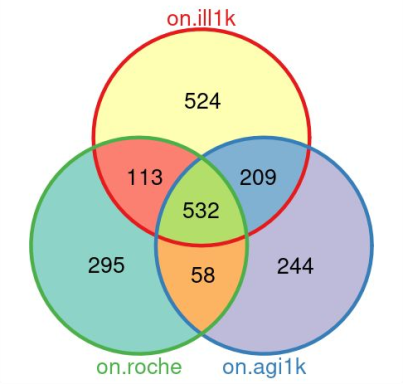
b**

**d**

in.DMETs in.HLAs in.MFisch in.lungXu in.bcRFus in.CMason in.breast in.GGrill

33 7 20 23 75 21 20 21

in.secFnd in.FDA

97 36

**Supplemental Figure 1: Comprehensive unified research panel design.** Our AGLR2 / ROCR2 research panel designs incorporate targets from **(a)** established onco-panels, labelled Qiagen, Thermo, xGen, ill170 [ILMR1], ill1k [ILMR2], agi151, agi1k[AGLR1], roche [ROCR1]. Comparisons of the three larger panels **(b)** and of the five smaller panels **(c)** showed that the panels were complementary. Our design included additional gene sets considered of interest to the community (d): FDA: approved cancer biomarkers; secFnd: recommended reports of secondary findings (AMA); bcRFus: fusions repeatedly observed in breast cancer; DMETs; HLAs; MFisch: neuroblastoma relevant; lungXu: lung cancer relevant; CMason: pharmagenomics genes annotated by the FDA (https://www.fda.gov/medical-devices/precision-medicine/table-pharmacogenetic-associations); breast: breast cancer relevant; GGrill: ageing/splicing relevant; and one gene relevant to skin cancer.
